# Supplementary material for: Coral bacterial community structure responds to environmental change in a host-specific manner
Source: Nat Commun. 2019 Jul 12;10:3092. doi: 10.1038/s41467-019-10969-5 (PMC6626051; doi:10.1038/s41467-019-10969-5)
Supplement: Supplementary file 1 — Supplementary Information [file 41467_2019_10969_MOESM1_ESM.pdf]

Supplementary Information

Coral bacterial community structure responds to environmental change in a host-specific manner

Maren Ziegler[1,2,8,+], Carsten GB Grupstra[1,3,8], Marcelle M. Barreto[1], Martin Eaton[4], Jaafar BaOmar[5,6,], Khalid Zubier[5], Abdulmohsin Al-Sofyani[5], Adnan J. Turki[5], Rupert Ormond[4,5,], Christian R. Voolstra[1,7,+]

[1] Red Sea Research Center, Division of Biological and Environmental Science and Engineering (BESE), 4700 King Abdullah University of Science and Technology (KAUST), Thuwal 23955, Saudi Arabia

[2] Department of Animal Ecology & Systematics, Justus Liebig University, Heinrich-Buff-Ring 26-32 IFZ, 35392 Giessen, Germany

[3] BioSciences Department, Rice University, 6100 Main Street, Houston, Texas 77005, USA

[4] Centre for Marine Biology and Biodiversity, Institute for Earth and Life Sciences, Heriot-Watt University, Riccarton, Edinburgh EH14 4AS, Scotland, United Kingdom

[5] Faculty of Marine Science, King Abdulaziz University, PO Box 80207, Jeddah 21589, Saudi Arabia

[6] Department of Marine Biology, Faculty of Environmental Science and Marine Biology, Hadhramout University, Al-Mukalla, Republic of Yemen.

[7] Department of Biology, University of Konstanz, 78457 Konstanz, Germany

[8] These authors contributed equally.

[+] Corresponding authors: maren.ziegler@bio.uni-giessen.de and christian.voolstra@uni-konstanz.de

Short title: Microbiome flexibility of corals

Supplementary Methods

Quantification of anthropogenic impacts

To characterize the anthropogenic impacts at our study sites, we quantified sediment loads, inorganic nitrate and total hydrocarbons over the period of one year. Specifically, sediment loads at the six sites (A-F) were determined using duplicate sediment traps, one attached in mid-water to each end of the coral culture rig. Each trap consisted of a vertically orientated 40 cm long x 10 cm diameter PVC cylinder, with two internal vertical baffles inserted to limit particle resuspension [1, 2]. At intervals of 2 - 6 months the traps were first capped (to prevent loss of sediment during transport) and then collected for return to the laboratory, where the sediment from each trap was filtered through 0.45um GF/C filter paper, dried at 80 ˚C for 8 h and then weighed. Concentrations of inorganic nitrate were determined according to the method recommended by Parsons *et al*., [3], using a Pye-Unicam Spectrophotometer (Model PU-8600).

Water samples were analyzed for total hydrocarbons (THCs), based on the method of Law, Fileman, and Portman [4] as adopted by ROMPE [5]. Extraction was carried out with 4 x 25 ml of dichloromethane (CH_2Cl_2) after drying over anhydrous sodium sulphate. The solvent was removed by rotary evaporation, and the extracts then taken up in *n*-hexane. Total hydrocarbons, in water extracts, were then measured by fluorescence spectrophotometry on a Shimadzu Dual Beam Difference Spectrofluorophotometer (Model RF-500), with the excitation wavelength at 310 nm and the emission scanned from 320 - 500 nm and quantified at 360 nm. Light Arabian crude oil (LAO) was used for standardization and quantification.

Supplementary Figures

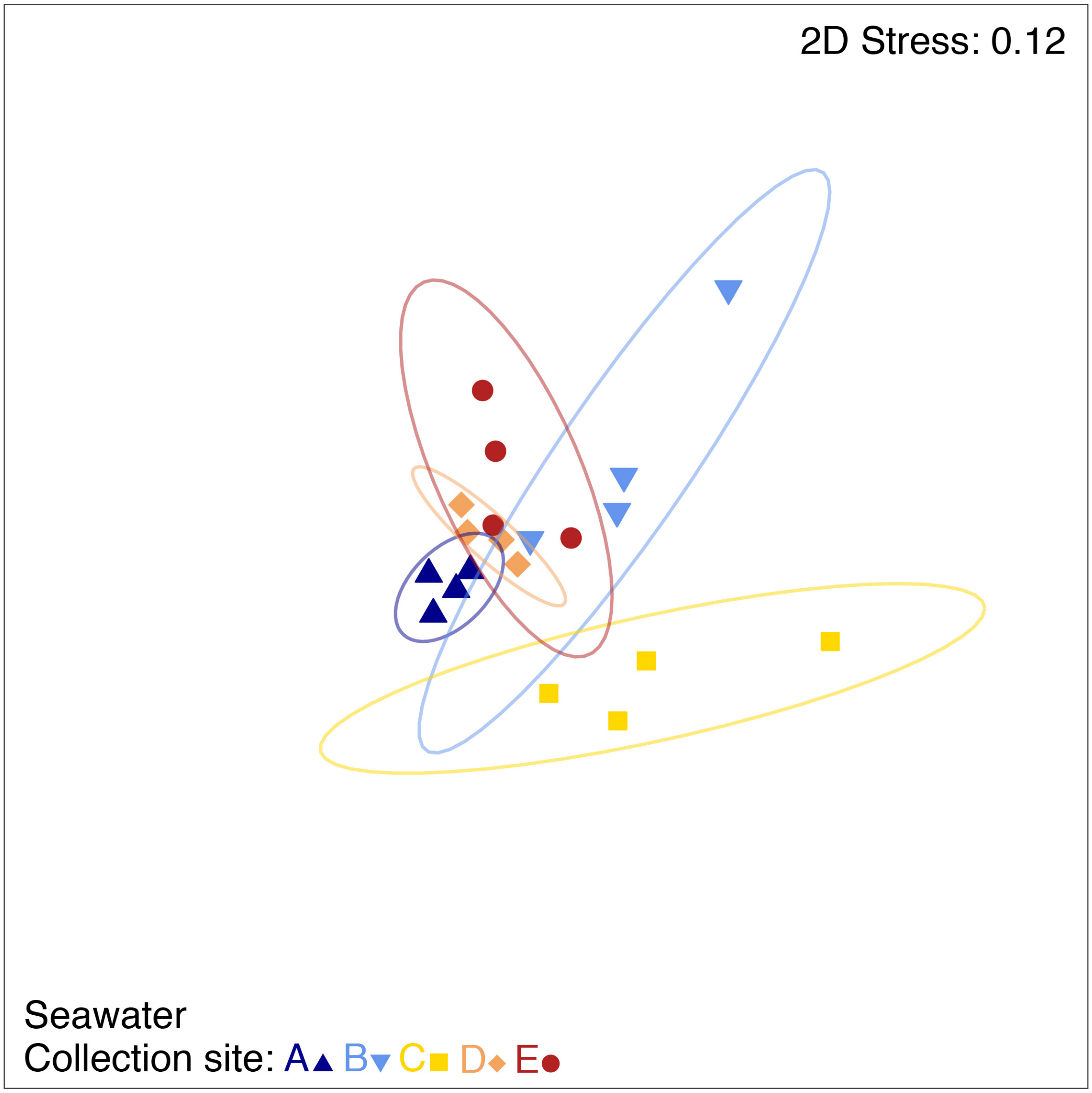

Supplementary Figure 1. Bacterial community structure and relative dispersion of seawater samples. Samples were taken from five sites close to the city of Jeddah in the Red Sea and are shown based on non-metric multidimensional scaling (nMDS) of Bray-Curtis distances. Ellipses denote 90 % confidence intervals. Source data are provided as a Source Data file.

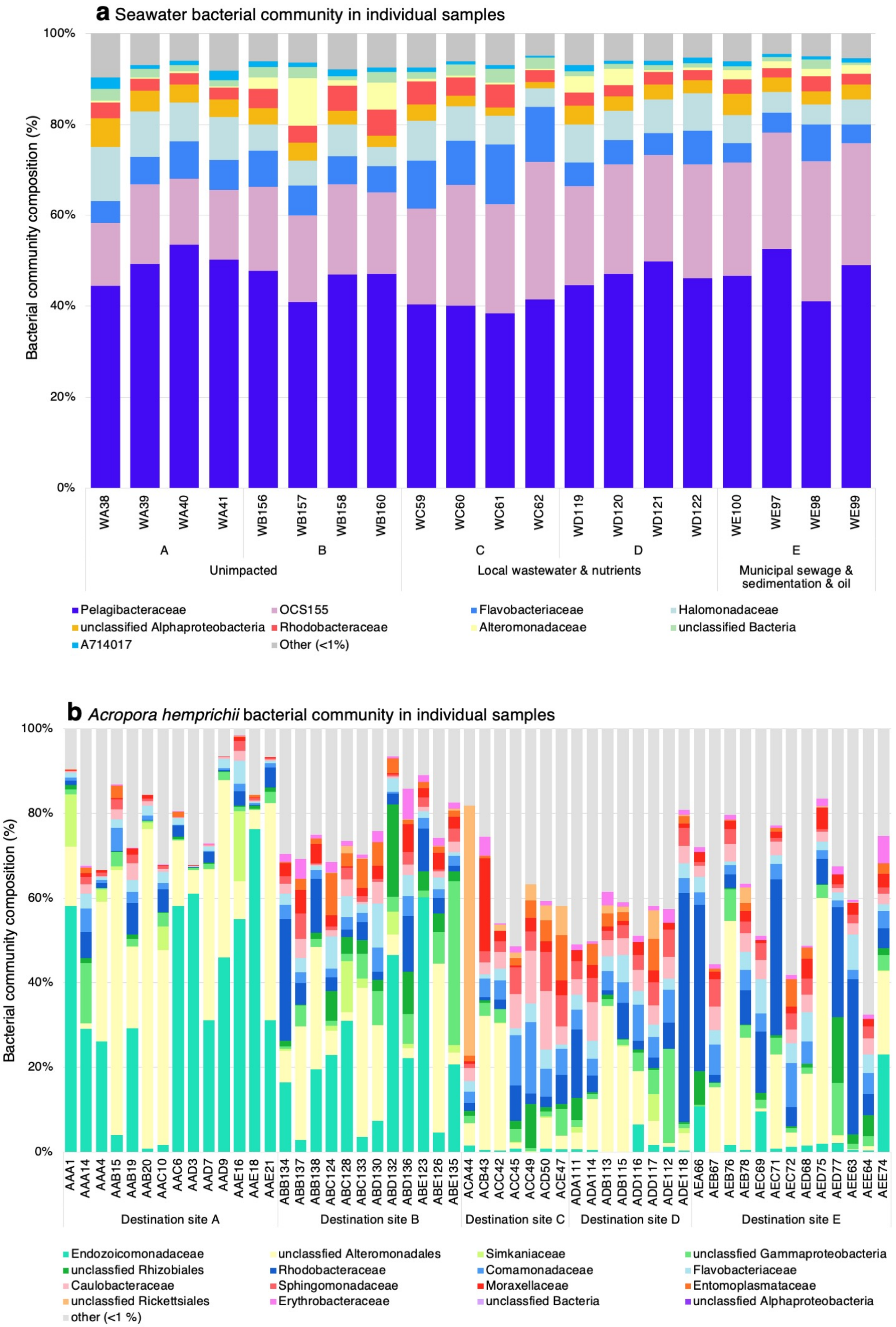
a Seawater bacterial community in individual samples
Bacterial community composition (%)
100%
80%
60%
40%
20%
0%
WA38
WA39
WA40
WA41
WB156
WB157
WB158
WB160
WC59
WC60
WC61
WC62
WD119
WD120
WD121
WD122
WE100
WE97
WE98
WE99
A
B
C
D
E
Unimpacted
Local wastewater & nutrients
Municipal sewage & sedimentation & oil
Pelagibacteraceae
OCS155
Flavobacteriaceae
Halomonadaceae
unclassified Alphaproteobacteria
Rhodobacteraceae
Alteromonadaceae
unclassified Bacteria
A714017
Other (<1%)
b Acropora hemprichii bacterial community in individual samples
Bacterial community composition (%)
100%
80%
60%
40%
20%
0%
AAA1
AAA14
AAA4
AAB15
AAB19
AAB20
AAC10
AAC6
AAD3
AAD7
AAD9
AAE16
AAE18
AAE21
ABB134
ABB137
ABB138
ABC124
ABC128
ABC133
ABD130
ABD132
ABD136
ABE123
ABE126
ABE135
ACA44
ACB43
ACC42
ACC45
ACC49
ACD50
ACE47
ADA111
ADA114
ADB113
ADB115
ADD116
ADD117
ADE112
ADE118
AEA66
AEB67
AEB76
AEB78
AEC69
AEC71
AEC72
AED68
AED75
AED77
AEE63
AEE64
AEE74
Destination site A
Destination site B
Destination site C
Destination site D
Destination site E
Endozoicomonadaceae
unclassfied Alteromonadales
Simkaniaceae
unclassfied Gammaproteobacteria
unclassfied Rhizobiales
Rhodobacteraceae
Comamonadaceae
Flavobacteriaceae
Caulobacteraceae
Sphingomonadaceae
Moraxellaceae
Entomoplasmataceae
unclassfied Rickettsiales
Erythrobacteraceae
unclassfied Bacteria
unclassfied Alphaproteobacteria
other (<1 %)

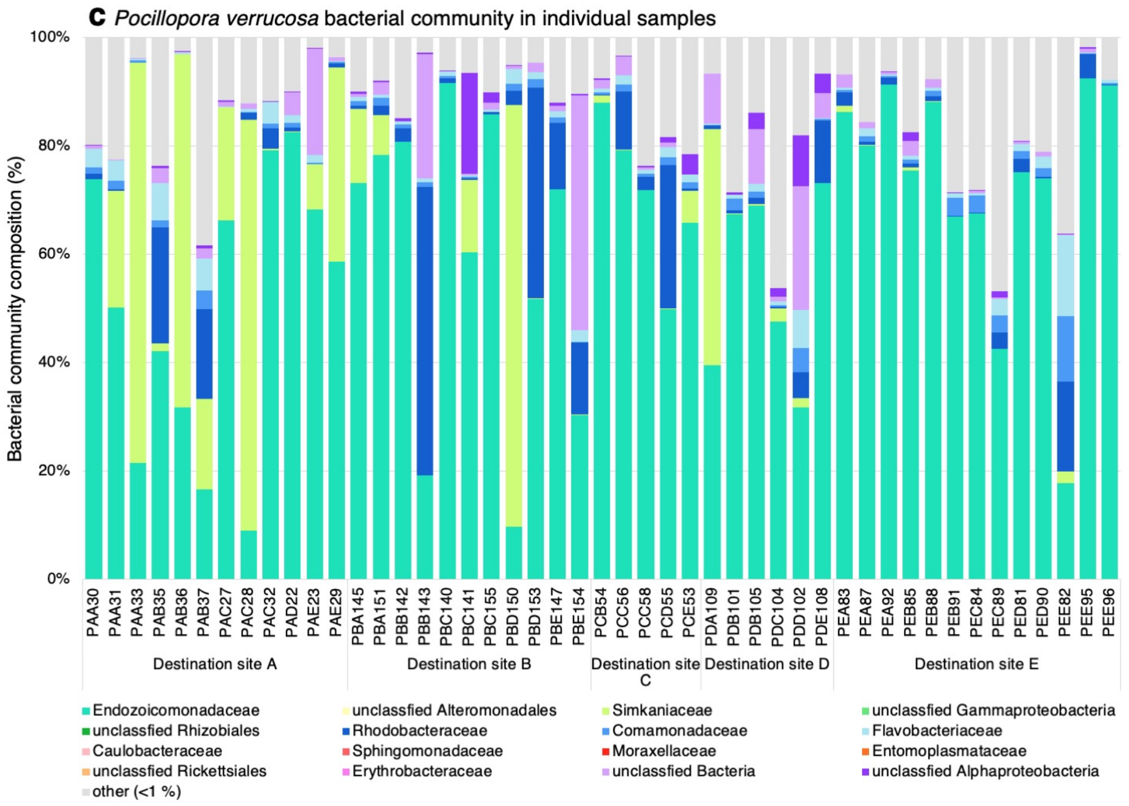

Supplementary Figure 2. Bacterial community composition in all individual samples. Bacterial community composition in samples of a) seawater, b) the coral *Acropora hemprichii*, and c) the coral *Pocillopora verrucosa* after a 21-months cross-transplantation experiment close to the city of Jeddah in the Red Sea. Samples denoted by destination site. Source data are provided as a Source Data file.

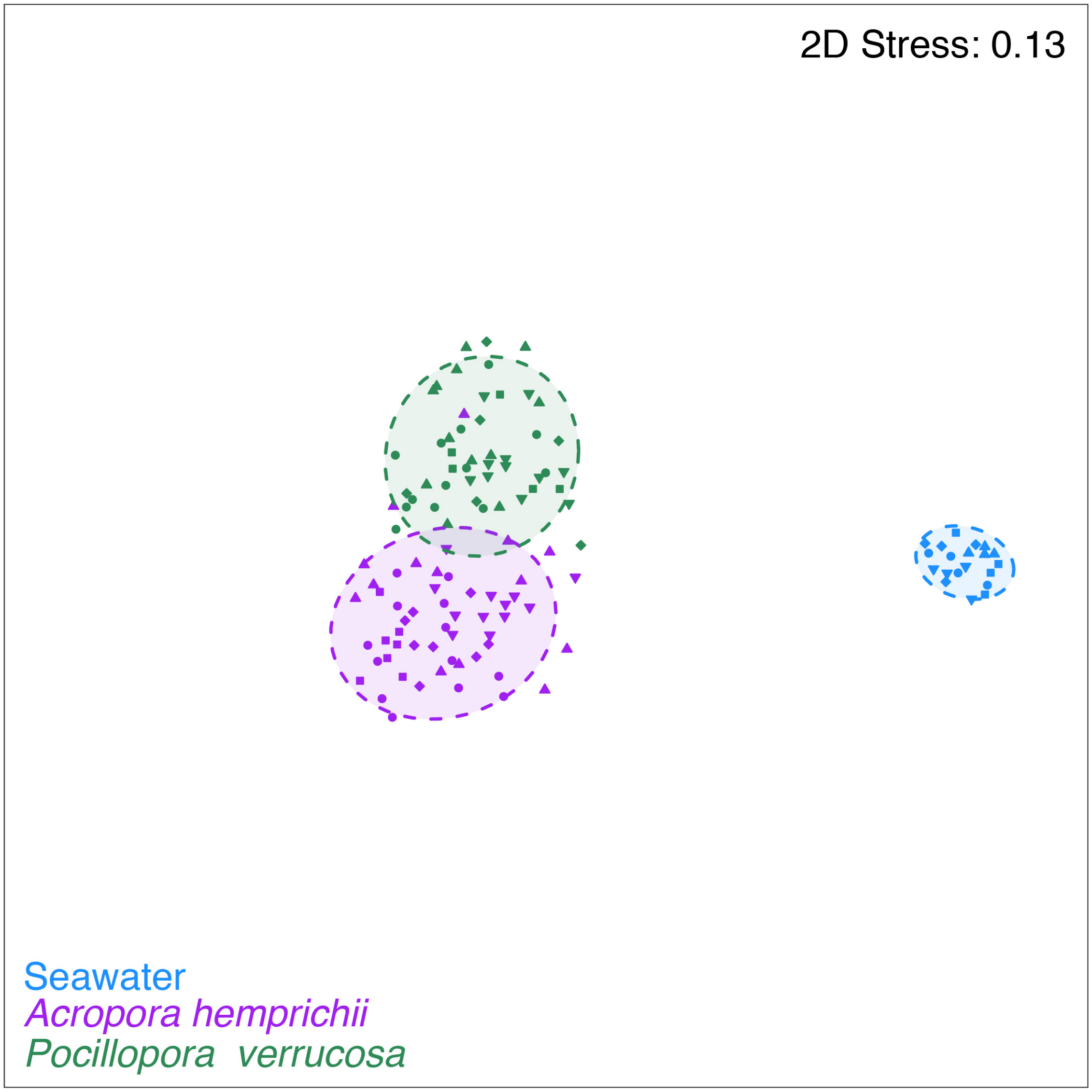

Supplementary Figure 3. Bacterial community structure and relative dispersion per sample type. Bacterial communities associated with the coral species *Acropora hemprichii* and *Pocillopora verrucosa* and seawater samples from five sites close to the city of Jeddah in the Red Sea (after 21 months of reciprocal transplantation) based on non-metric multidimensional scaling (nMDS) of Bray-Curtis distances**.** Ellipses denote 90 % confidence intervals. Source data are provided as a Source Data file.

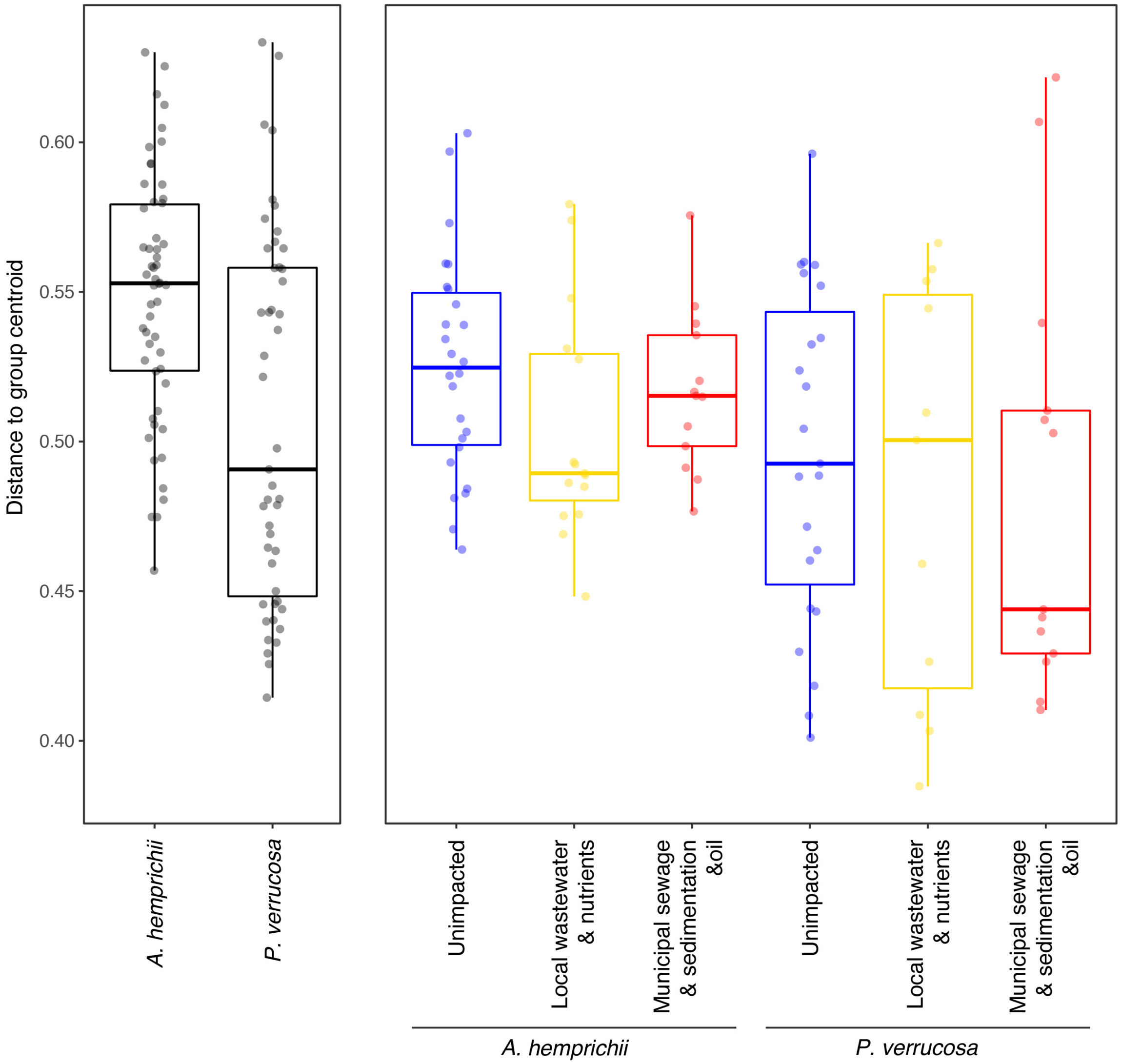

Supplementary Figure 4. Multivariate dispersion of microbial community samples. Bacterial community dispersion from the coral species *Acropora hemprichii* and *Pocillopora verrucosa* after 21months of reciprocal transplantation between 3 levels of anthropogenic impact (colors: blue = unimpacted, yellow = local wastewater & nutrients, red = municipal wastewater & sedimentation & oil) close to the city of Jeddah in the Red Sea. Microbial communities of *A. hemprichii* are significantly more variable than those of *P. verrucosa* (left graph, ANOVA, F = 16.01, $p < 0.001$), while differences between destination impacts within species were not significantly different (right graph, ANOVA, all $p > 0.05$). Boxes represent the 25th to 75th percentile, lines show medians, error bars represent smallest/largest values to a maximum of 1.5 * IQR. Source data are provided as a Source Data file.

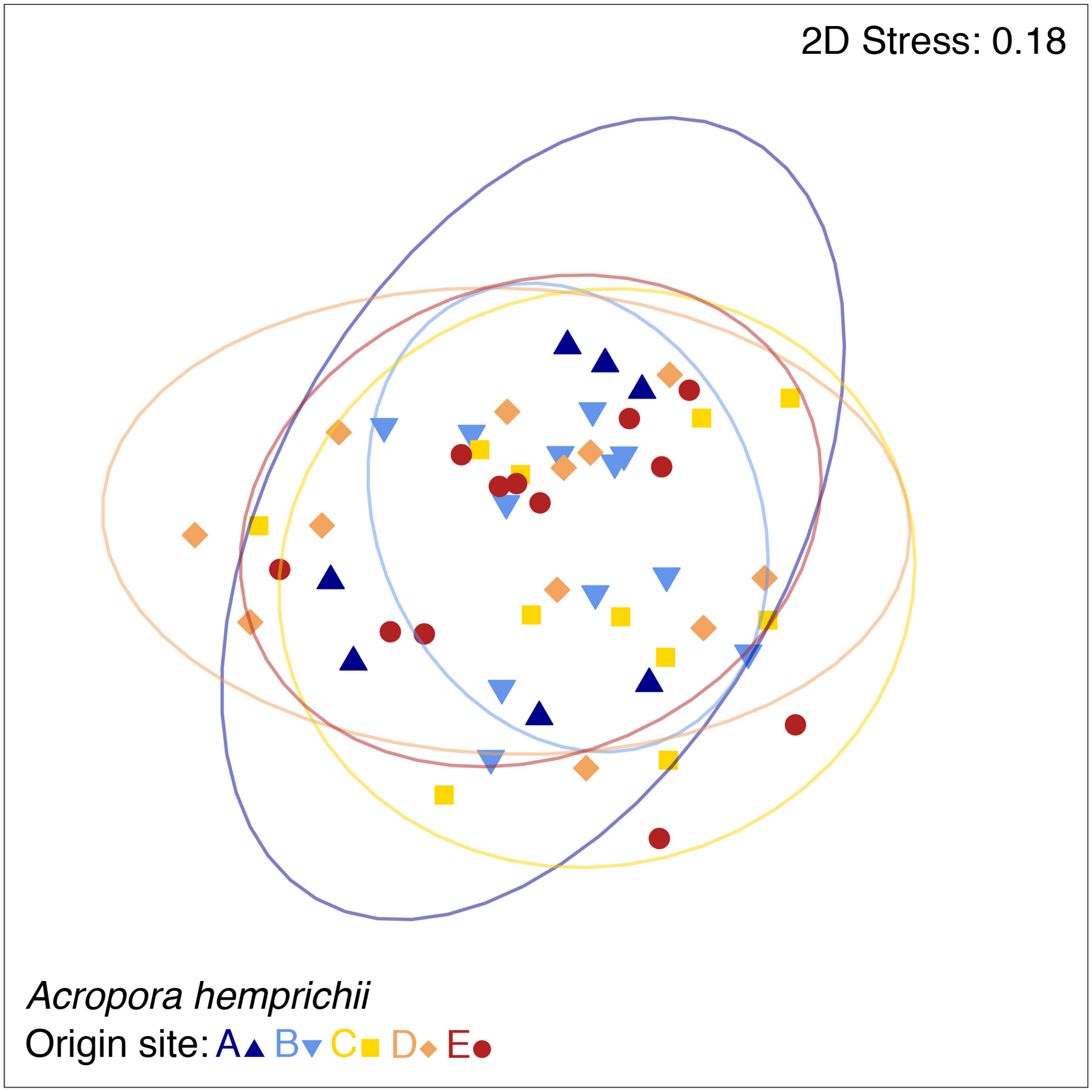

Supplementary Figure 5. Bacterial community structure and relative dispersion in *A. hemprichii.* Data shown based on non-metric multidimensional scaling (nMDS) of Bray-Curtis distances. Data shown as site of origin after 21 months of reciprocal transplantation. A/B unimpacted sites; C/D local wastewater & nutrients; E municipal sewage & sedimentation & oil. Ellipses denote 90 % confidence intervals. Source data are provided as a Source Data file.

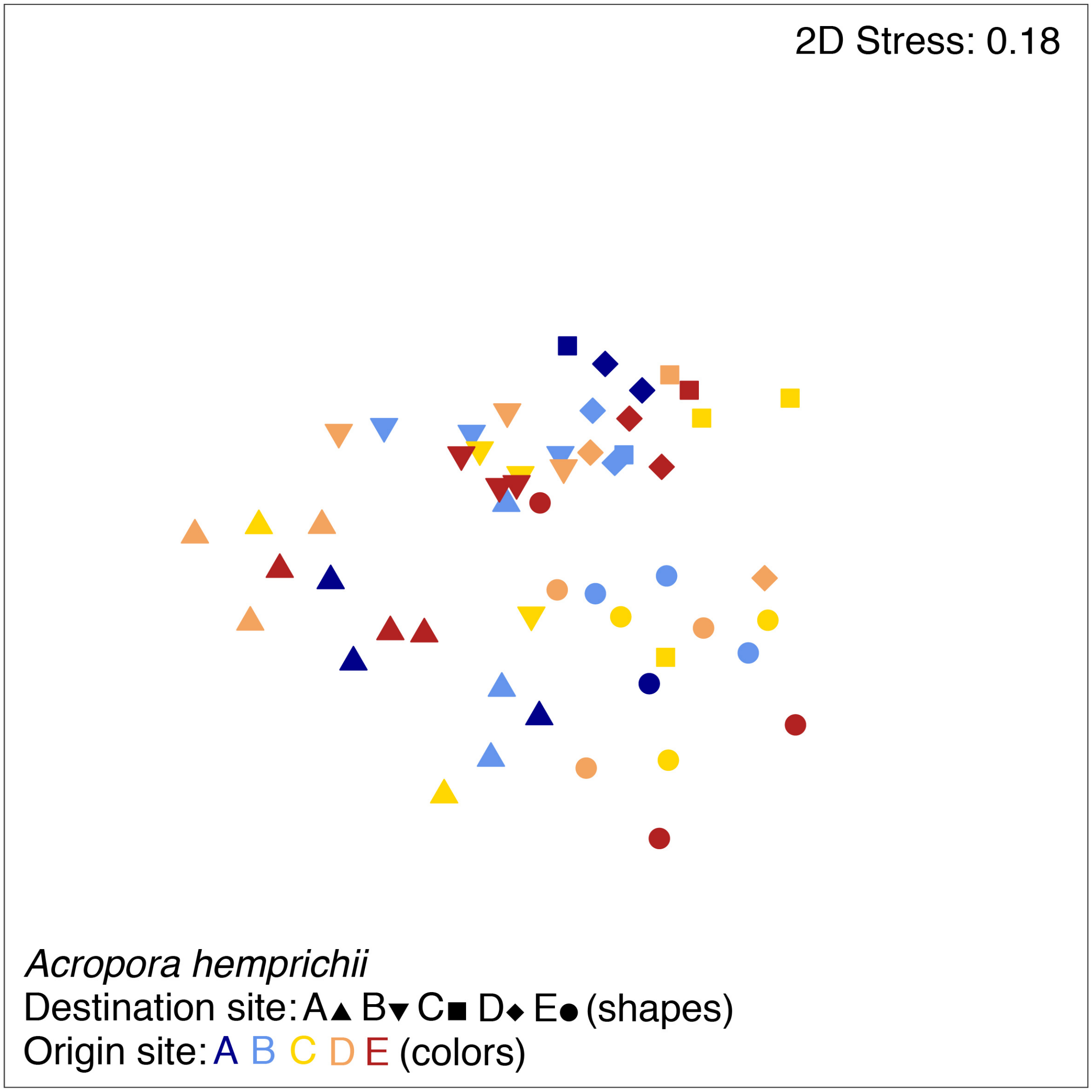

Supplementary Figure 6. Bacterial community structure in *A. hemprichii.* Data shown based on non-metric multidimensional scaling (nMDS) of Bray-Curtis distances. Data shown as site of destination (shapes) and site of origin (colors) after 21 months of reciprocal transplantation. A/B unimpacted sites; C/D local wastewater & nutrients; E municipal sewage & sedimentation & oil. Source data are provided as a Source Data file.

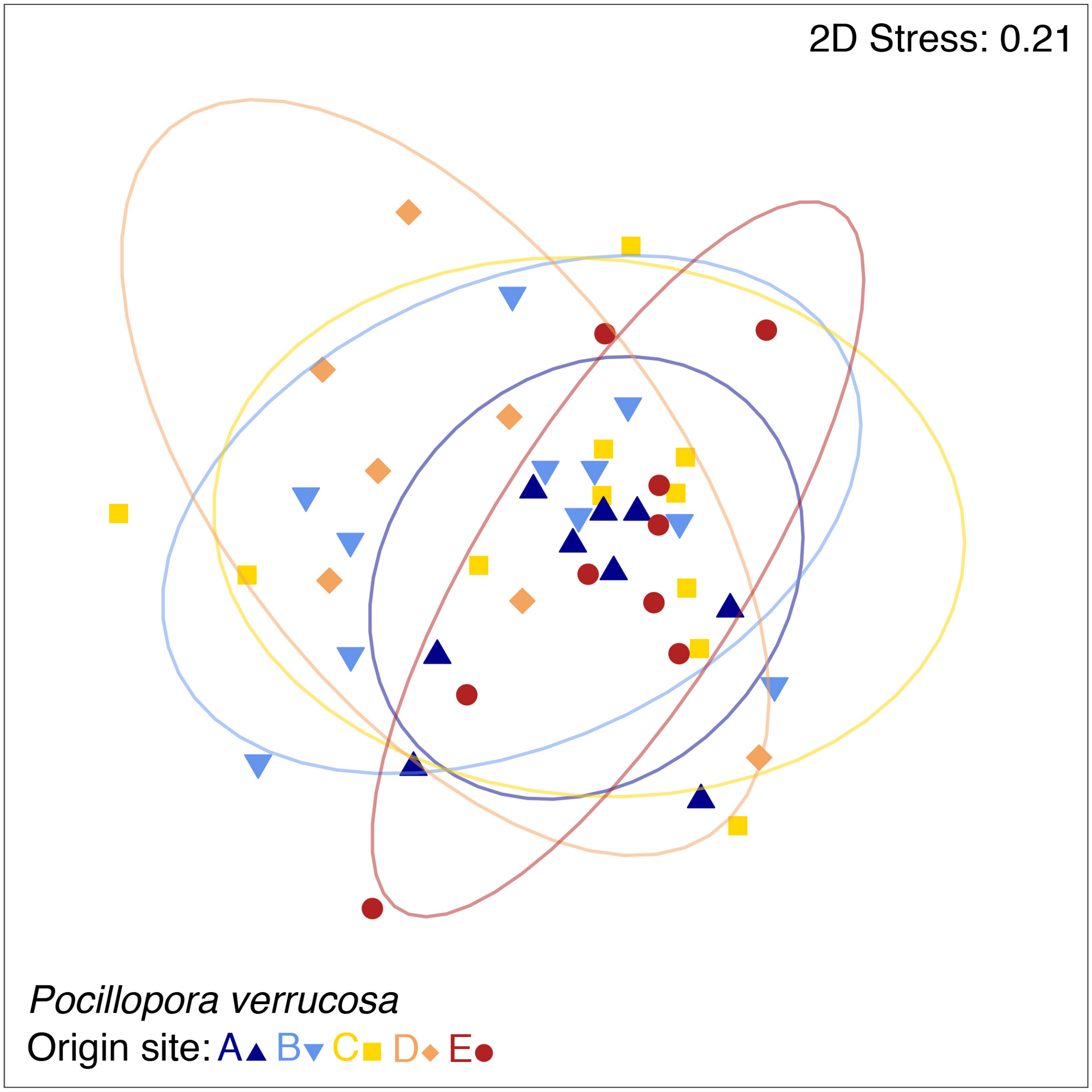

Supplementary Figure 7. Bacterial community structure and relative dispersion in *P. verrucosa.* Data shown based on non-metric multidimensional scaling (nMDS) of Bray-Curtis distances. Data shown as site of origin after 21 months of reciprocal transplantation. A/B unimpacted sites; C/D local wastewater & nutrients; E municipal sewage & sedimentation & oil. Ellipses denote 90 % confidence intervals. Source data are provided as a Source Data file.

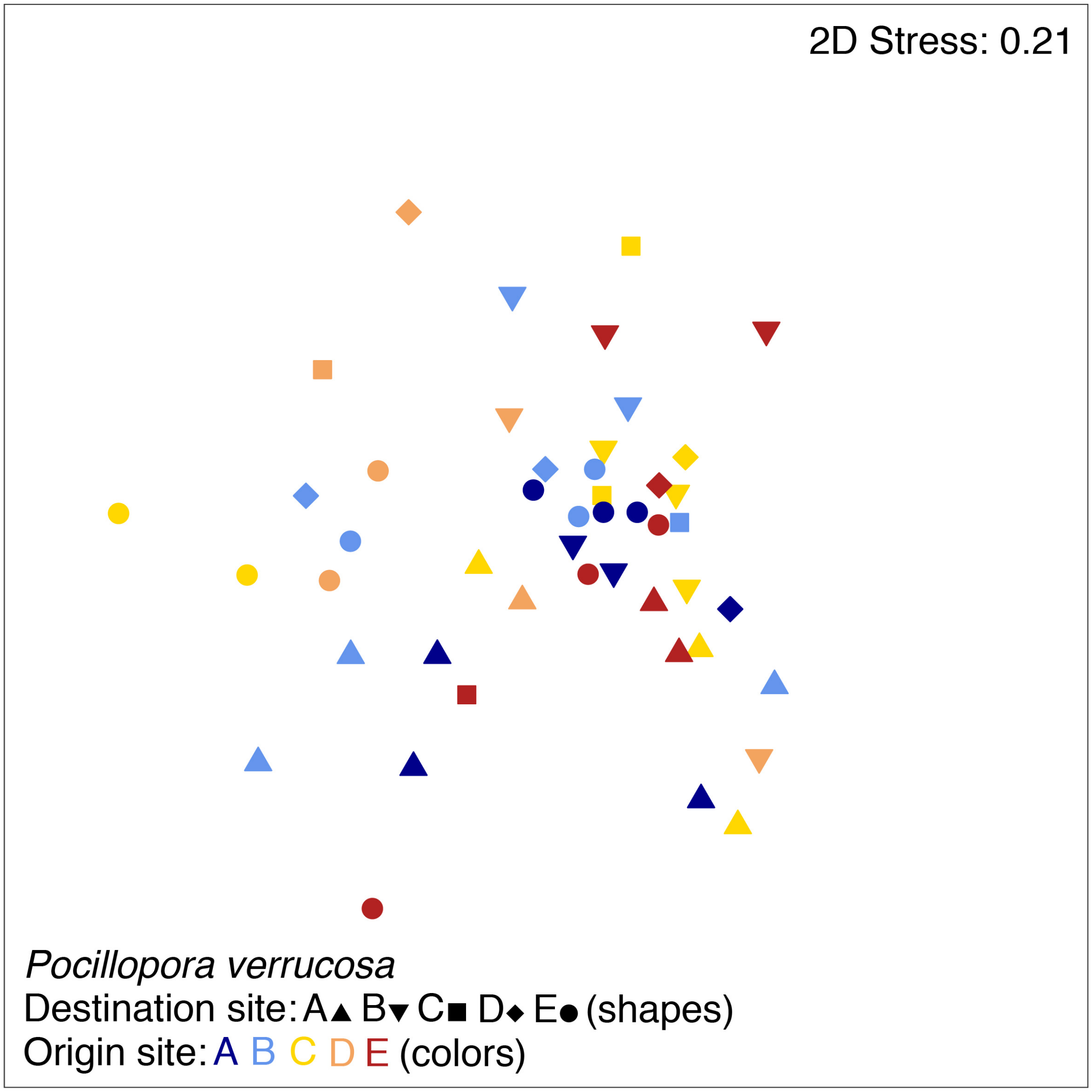

Supplementary Figure 8. Bacterial community structure in *P. verrucosa.* Data shown based on non-metric multidimensional scaling (nMDS) of Bray-Curtis distances. Data shown as site of destination (shapes) and site of origin (colors) after 21 months of reciprocal transplantation. A/B unimpacted sites; C/D local wastewater & nutrients; E municipal sewage & sedimentation & oil. Source data are provided as a Source Data file.

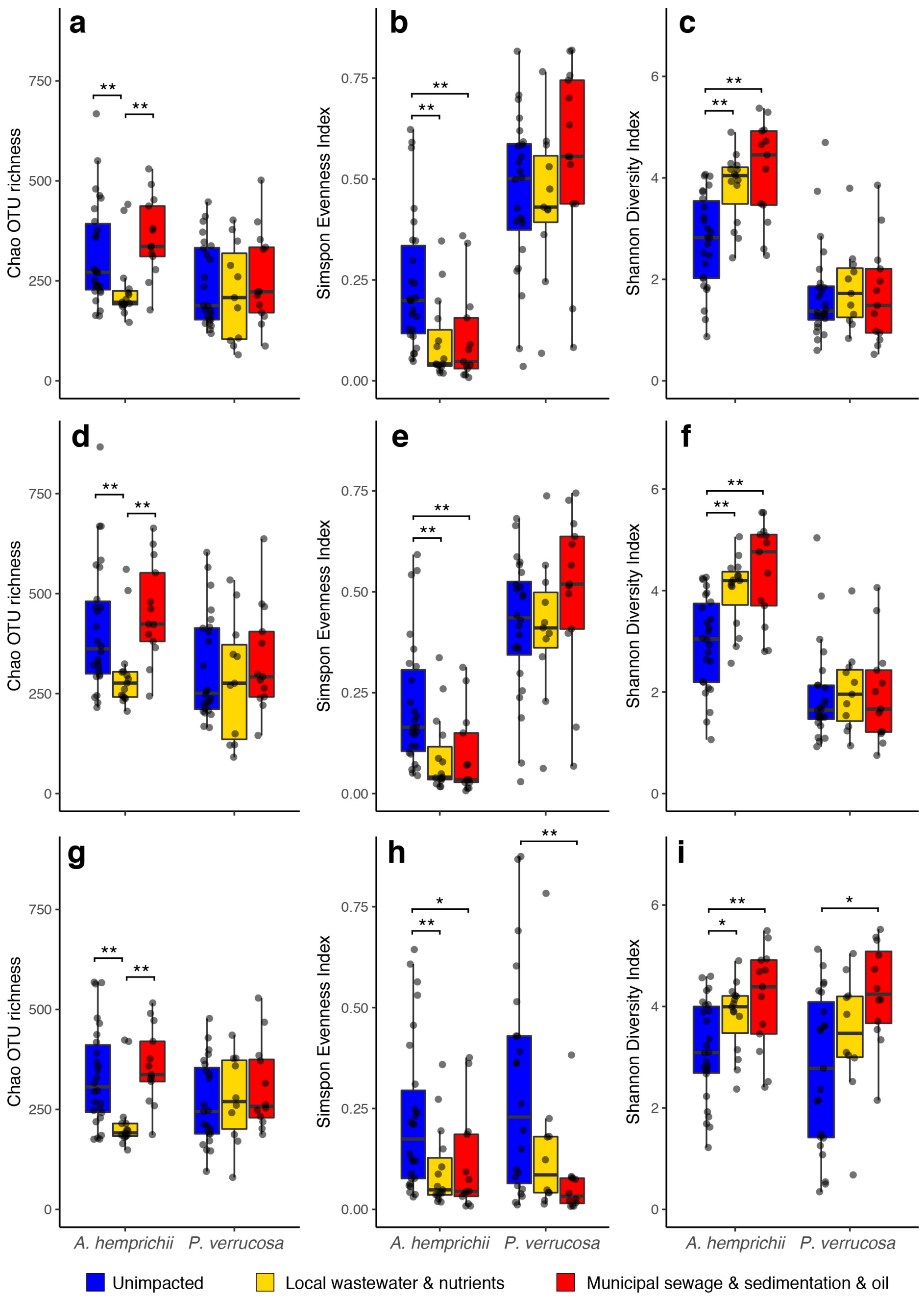

Supplementary Figure 9. Bacterial richness and diversity of Red Sea corals. Fragments of the corals *Acropora hemprichii* and *Pocillopora verrucosa* were exposed to different levels of anthropogenic impact near Jeddah, Red Sea. Samples were collected after 21 months of reciprocal transplantation across different impacts and are displayed by destination impact. Different rows represent separate data analyses: **(a – c)** 97 % OTU data, **(d – f)** 99 % OTU data, **(g – i)** 97 % OTU data without Endozoicomonadaceae lineage. Boxes represent the 25th to 75th percentile, lines show medians, error bars represent smallest/largest values to a maximum of 1.5 * IQR, Kruskal_Wallis test significance levels: * $p < 0.05$, ** $p < 0.005$. Source data are provided as a Source Data file.

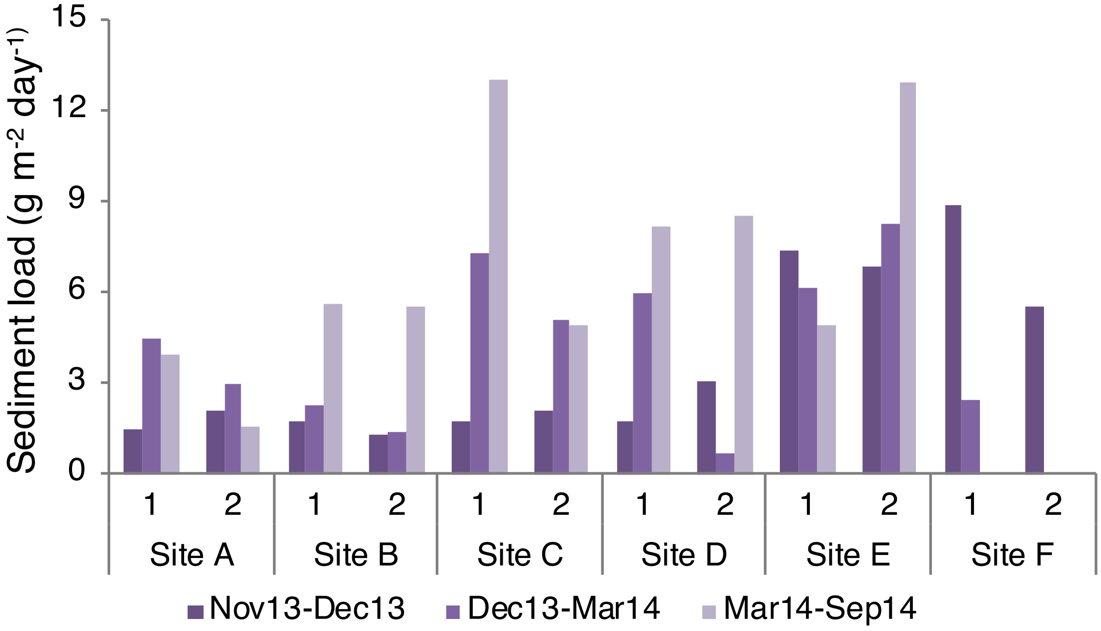

Supplementary Figure 10. Sediment load at six reef sites at Jeddah. Measurements were taken during three deployment periods (colors) and determined from duplicate sediment traps (shown separately) at six reef sites close to the city of Jeddah in the Red Sea. Site F including all corals and sediment traps was lost prior to the last collection in September 2014, and replicate 2 at site F was lost during the second deployment (missing data). Source data are provided as a Source Data file.

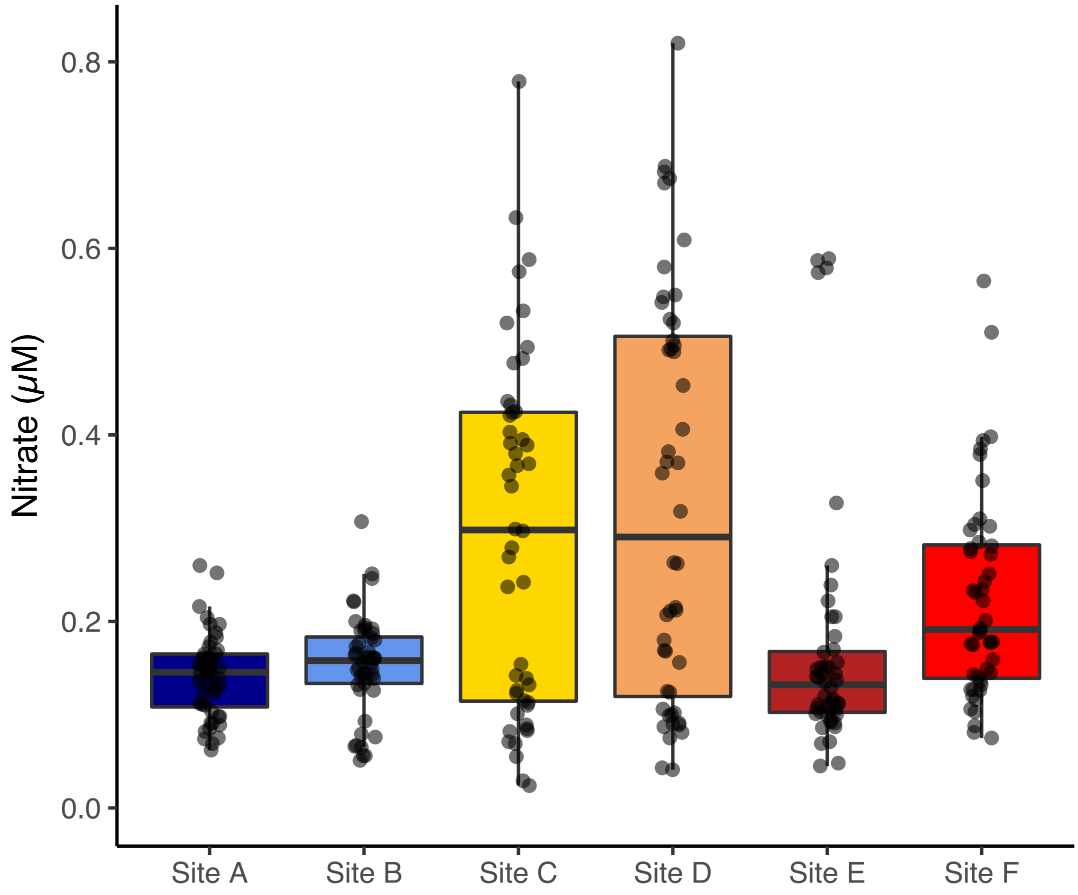

Supplementary Figure 11. Dissolved inorganic nitrate at six reef sites at Jeddah. Measurements were taken in the Red Sea between October 2013 – September 2014. Four measurements were taken each month, one each per depth (1, 5, 10, 20 m). Boxes represent the 25th to 75th percentile, lines show medians, error bars represent smallest/largest values to a maximum of 1.5 * IQR. Source data are provided as a Source Data file.

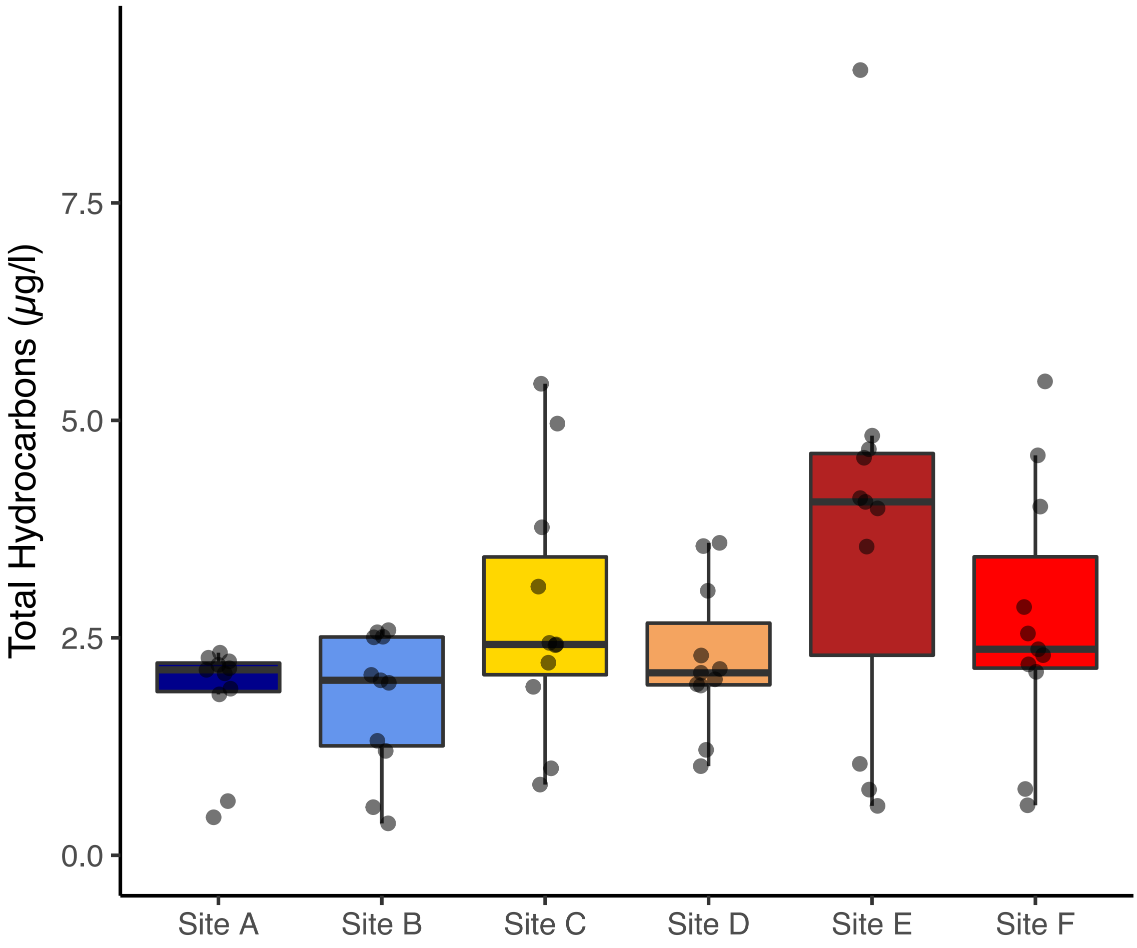

Supplementary Figure 12. Concentration of total hydrocarbons (µg L^{-1}) at six reef sites at Jeddah. Samples were collected monthly from 1 m below the surface between October 2013 – September 2014 and measurements were standardized against Light Arabian Oil. Boxes represent the 25th to 75th percentile, lines show medians, error bars represent smallest/largest values to a maximum of 1.5 * IQR. Source data are provided as a Source Data file.

Supplementary Table

Supplementary Table 1. Core microbiome of two Red Sea coral species. List of members of the bacterial core microbiome for the corals *Acropora hemprichii* and *Pocillopora verrucosa* after a 21-months cross-transplantation experiment at reef sites close to the city of Jeddah in the Red Sea. OTUs were considered part of the core microbiome if they were identified in at least 75 % of samples at every site. The highest level of annotation is provided for each OTU, followed by the level of that classification.

**Core microbiome of *Acropora hemprichii***		**Fraction of corals with OTU at each destination site (A - E)**					**Number of sequences per sample at each destination site as means (SD)**				
**OTU**	**Greengenes annot. (taxonomic level)**	**A**	**B**	**C**	**D**	**E**	**A**	**B**	**C**	**D**	**E**
Otu0002	*Melitea* sp. (g)	1.00	1.00	0.86	1.00	0.77	991 (704)	558 (521)	348 (430)	502 (428)	567 (633)
**Otu0007**	***Caulobacter* sp. (g)**	1.00	1.00	1.00	1.00	1.00	30 (34)	64 (49)	187 (111)	155 (67)	100 (37)
**Otu0014**	***Sphingomonas* sp. (g)**	0.93	1.00	0.86	1.00	1.00	16 (20)	36 (44)	90 (80)	79 (47)	42 (33)
**Otu0017**	***Flavobacterium* sp. (g)**	1.00	1.00	1.00	1.00	1.00	16 (16)	20 (11)	30 (25)	57 (28)	47 (24)
Otu0021	Unclassified Comamonadaceae (f)	0.86	1.00	0.86	1.00	0.92	8 (11)	22 (10)	33 (22)	48 (32)	26 (16)
Otu0026	*Pelomonas* sp. (g)	1.00	0.92	1.00	0.88	1.00	7 (7)	11 (7)	32 (26)	32 (18)	29 (15)
Otu0031	*Staphylococcus* sp. (g)	0.86	0.83	0.86	0.88	1.00	5 (6)	15 (18)	34 (45)	22 (21)	20 (22)

**Core microbiome of *Pocillopora verrucosa***		**Fraction of corals with OTU at a site (A - E)**					**Number of sequences per sample at a site as means (SD)**				
**OTU**	**Greengenes annot. (taxonomic level)**	**A**	**B**	**C**	**D**	**E**	**A**	**B**	**C**	**D**	**E**
Otu0001	Unclassified Endozoicomonadaceae (f)	1.00	1.00	1.00	1.00	1.00	1,427 (809)	1,756 (842)	2,045 (526)	2,225 (665)	2,220 (647)
Otu0003	Unclassified Endozoicomonadaceae (f)	0.92	1.00	1.00	0.83	1.00	52 (67)	53 (53)	20 (23)	28 (21)	42 (32)
**Otu0007**	***Caulobacter* sp. (g)**	1.00	0.91	1.00	1.00	1.00	17 (22)	18 (14)	22 (11)	41 (45)	30 (27)
Otu0011	*Erythrobacter* sp. (g)	0.92	1.00	1.00	1.00	1.00	8 (13)	32 (20)	13 (12)	79 (84)	24 (19)
**Otu0014**	***Sphingomonas* sp. (g)**	0.85	0.91	0.80	0.83	1.00	9 (11)	7 (8)	6 (3)	8 (6)	12 (12)
**Otu0017**	**Flavobacterium sp. (g)**	0.85	1.00	1.00	1.00	0.92	13 (15)	60 (2)	13 (3)	10 (9)	27 (55)

Supplementary References

1. Storlazzi, C. D., Field, M. E., Bothner, M. H. The use (and misuse) of sediment traps in coral reef environments: theory, observations, and suggested protocols. *Coral Reefs* **30**, 23-38 (2010).

2. Gardner, W. D. Field assessment of sediment traps. *J Mar Research* **38**, 41-52 (1980).

3. Parsons, T. R., Maita, Y., Lalli, C. M. *A Manual of Chemical and Biological Methods for Seawater Analysis*. Pergamon Press (1984).

4. Law, R. J., Fileman, T. W., Portman, J. E. Methods of analysis of hydrocarbons in marine and other samples (Report No. 2). Ministry of Agriculture Fisheries and Food (1988).

5. ROPME. *Manual of Oceanography Observation and Pollution Analysis Methods*. Regional Organization for the Protection of the Marine Environment (1989).
