# Supplementary material for: Coral bacterial community structure responds to environmental change in a host-specific manner
Source: Nat Commun. 2019 Jul 12;10:3092. doi: 10.1038/s41467-019-10969-5 (PMC6626051; doi:10.1038/s41467-019-10969-5)
Supplement: Supplementary file 3 — Description of Additional Supplementary Files [file 41467_2019_10969_MOESM3_ESM.pdf]

Description of Additional Supplementary Files

File Name: Supplementary Data 1
Description: Number of sequences available per sample before and after quality control.

File Name: Supplementary Data 2
Description: Representative sequences for bacterial OTUs at 97 % similarity cutoff.

File Name: Supplementary Data 3
Description: Statistical results (PERMANOVA) of re-analyses of data sets at 99 % similarity cutoff for OTUs, at 97 % similarity cutoff excluding Endozoicomonadaceae family, and between impacts (pooled sites) for all data sets.

File Name: Supplementary Data 4
Description: Count table of bacterial OTUs at 99 % similarity cutoff after subsampling to 3,101 sequences per sample.

File Name: Supplementary Data 5
Description: Count table of bacterial OTUs at 97 % similarity cutoff without Endozoicomonadaceae family after subsampling to 3,101 sequences per sample.

File Name: Supplementary Data 6
Description: Indicator species analysis of bacterial taxa associated with the corals Acropora hemprichii and Pocillopora verrucosa between sites close to Jeddah, Red Sea.
