# Supplementary material for: Coral bacterial community structure responds to environmental change in a host-specific manner
Source: Nat Commun. 2019 Jul 12;10:3092. doi: 10.1038/s41467-019-10969-5 (PMC6626051; doi:10.1038/s41467-019-10969-5)
Supplement: Supplementary file 10 — Reporting Summary [file 41467_2019_10969_MOESM10_ESM.pdf]

nature research

Corresponding author(s): Christian R Voolstra, Maren Ziegler

Last updated by author(s): May 21, 2019

# Reporting Summary

Nature Research wishes to improve the reproducibility of the work that we publish. This form provides structure for consistency and transparency in reporting. For further information on Nature Research policies, see Authors & Referees and the Editorial Policy Checklist.

## Statistics

For all statistical analyses, confirm that the following items are present in the figure legend, table legend, main text, or Methods section.

| n/a | Confirmed | |
|---|---|---|
| ☐ | ☒ | The exact sample size (*n*) for each experimental group/condition, given as a discrete number and unit of measurement |
| ☐ | ☒ | A statement on whether measurements were taken from distinct samples or whether the same sample was measured repeatedly |
| ☐ | ☒ | The statistical test(s) used AND whether they are one- or two-sided<br>*Only common tests should be described solely by name; describe more complex techniques in the Methods section.* |
| ☒ | ☐ | A description of all covariates tested |
| ☐ | ☒ | A description of any assumptions or corrections, such as tests of normality and adjustment for multiple comparisons |
| ☐ | ☒ | A full description of the statistical parameters including central tendency (e.g. means) or other basic estimates (e.g. regression coefficient) AND variation (e.g. standard deviation) or associated estimates of uncertainty (e.g. confidence intervals) |
| ☐ | ☒ | For null hypothesis testing, the test statistic (e.g. *F*, *t*, *r*) with confidence intervals, effect sizes, degrees of freedom and *P* value noted<br>*Give P values as exact values whenever suitable.* |
| ☒ | ☐ | For Bayesian analysis, information on the choice of priors and Markov chain Monte Carlo settings |
| ☒ | ☐ | For hierarchical and complex designs, identification of the appropriate level for tests and full reporting of outcomes |
| ☒ | ☐ | Estimates of effect sizes (e.g. Cohen's *d*, Pearson's *r*), indicating how they were calculated |

*Our web collection on statistics for biologists contains articles on many of the points above.*

## Software and code

Policy information about availability of computer code

| | |
|---|---|
| Data collection | All data analyses were performed in R version 3.5.0. No custom code was used. |
| Data analysis | All data analyses were performed in R version 3.5.0. No custom code was used. |

For manuscripts utilizing custom algorithms or software that are central to the research but not yet described in published literature, software must be made available to editors/reviewers. We strongly encourage code deposition in a community repository (e.g. GitHub). See the Nature Research guidelines for submitting code & software for further information.

## Data

Policy information about availability of data

All manuscripts must include a data availability statement. This statement should provide the following information, where applicable:

- Accession codes, unique identifiers, or web links for publicly available datasets
- A list of figures that have associated raw data
- A description of any restrictions on data availability

Sequence data determined in this study are available under NCBI BioProject ID PRJNA491299 (https://www.ncbi.nlm.nih.gov/bioproject/PRJNA491299). Abundant coral bacterial microbiome OTU reference sequences are available under GenBank Accession numbers MK736129 - MK736265 (https://www.ncbi.nlm.nih.gov/nuccore/?term=MK736129:MK736265[accn]) for Acropora hemprichii and under GenBank Accession numbers MK736048 - MK736100 (https://www.ncbi.nlm.nih.gov/nuccore/?term=MK736048:MK736100[accn]) for Pocillopora verrucosa. Source data underlying the figures, Supplementary Information, and statistical analyses are provided as a Source Data File.

# Field-specific reporting

Please select the one below that is the best fit for your research. If you are not sure, read the appropriate sections before making your selection.

☒ Life sciences ☐ Behavioural & social sciences ☐ Ecological, evolutionary & environmental sciences

For a reference copy of the document with all sections, see nature.com/documents/nr-reporting-summary-flat.pdf

# Life sciences study design

All studies must disclose on these points even when the disclosure is negative.

| | |
|---|---|
| Sample size | 155 samples were collected, of which 131 were successfully amplified. |
| Data exclusions | One sample (Sample ID: PAE24) was excluded from statistical analyses, as the microbial community values was an outlier for all metrics tested. |
| Replication | The replication of the study design is detailed in table 1 of the manuscript. The samples were retrieved at one time point after 21 months of reciprocal transplantation. |
| Randomization | Each coral colony was back- and cross-transplanted to control for effect of colony identity during transplantation. |
| Blinding | Sequence data QC was conducted with an automated script and the following statistical data analyses with sample IDs. |

# Reporting for specific materials, systems and methods

We require information from authors about some types of materials, experimental systems and methods used in many studies. Here, indicate whether each material, system or method listed is relevant to your study. If you are not sure if a list item applies to your research, read the appropriate section before selecting a response.

### Materials & experimental systems

| n/a | Involved in the study |
|---|---|
| ☒ | ☐ Antibodies |
| ☒ | ☐ Eukaryotic cell lines |
| ☒ | ☐ Palaeontology |
| ☐ | ☒ Animals and other organisms |
| ☒ | ☐ Human research participants |
| ☒ | ☐ Clinical data |

### Methods

| n/a | Involved in the study |
|---|---|
| ☒ | ☐ ChIP-seq |
| ☒ | ☐ Flow cytometry |
| ☒ | ☐ MRI-based neuroimaging |

## Animals and other organisms

Policy information about studies involving animals; ARRIVE guidelines recommended for reporting animal research

| | |
|---|---|
| Laboratory animals | NA |
| Wild animals | All coral colonies were collected in the field and cross-transplanted in a field-based experiment. At the end of the study the fragments were collected and frozen for sample processing. |
| Field-collected samples | All coral colonies were collected in the field and cross-transplanted in a field-based experiment. At the end of the study the fragments were collected and frozen for sample processing. |
| Ethics oversight | The Saudi Arabian coastguard authority issued sailing permits to the reef sites. No further approvals are required for work with coral animals. |

Note that full information on the approval of the study protocol must also be provided in the manuscript.
